# Knowledge and Acceptance of COVID-19 Vaccination among Undergraduate Students from Central and Southern Italy

**DOI:** 10.3390/vaccines9060638

**Published:** 2021-06-10

**Authors:** Francesca Gallè, Elita Anna Sabella, Paolo Roma, Osvalda De Giglio, Giuseppina Caggiano, Silvio Tafuri, Giovanna Da Molin, Stefano Ferracuti, Maria Teresa Montagna, Giorgio Liguori, Giovanni Battista Orsi, Christian Napoli

**Affiliations:** 1 Department of Movement Sciences and Wellbeing, University of Naples “Parthenope”, 80133 Naples, Italy; giorgio.liguori@uniparthenope.it; 2 Inter-University Research Centre “Population, Environment and Health”, University of Bari Aldo Moro, 70121 Bari, Italy; elita.sabella@uniba.it (E.A.S.); giovanna.damolin@uniba.it (G.D.M.); 3 Department of Human Neurosciences, “Sapienza” University of Rome, 00185 Rome, Italy; paolo.roma@uniroma1.it (P.R.); stefano.ferracuti@uniroma1.it (S.F.); 4 Department of Biomedical Science and Human Oncology, University of Bari Aldo Moro, 70124 Bari, Italy; osvalda.degiglio@uniba.it (O.D.G.); giuseppina.caggiano@uniba.it (G.C.); silvio.tafuri@uniba.it (S.T.); mariateresa.montagna@uniba.it (M.T.M.); 5 Department of Public Health and Infectious Diseases, “Sapienza” University of Rome, 00185 Rome, Italy; giovanni.orsi@uniroma1.it; 6 Department of Medical Surgical Sciences and Translational Medicine, “Sapienza” University of Rome, 00189 Rome, Italy; christian.napoli@uniroma1.it

**Keywords:** SARS-COV-2, COVID-19, vaccine hesitancy, vaccine acceptance, information, undergraduates

## Abstract

At the end of 2020, the Italian Ministry of Health launched a national vaccination campaign to counteract the COVID-19 pandemic. The present study aimed at appraising levels of knowledge about and acceptance of COVID-19 vaccination in a sample of Italian undergraduates during the first phase of the immunization plan. A web-based questionnaire was administered to students attending universities in Bari, Naples, and Rome during the period February–April 2021. Of the total of 3226 participants, 91.9% were keen to receive a COVID-19 vaccination. More than 80% gave correct answers to questions about COVID-19 vaccine administration, functioning, and effects on community health. However, only 63.8% identified the correct composition of the available vaccines. Knowledge was found to be related to sociodemographic features and COVID-19 vaccination acceptance (*p* < 0.05). COVID-19 vaccination acceptance was found to be related to having a previous vaccination against influenza (OR 3.806, CI 95% 1.181–12.267; *p* = 0.025) and knowledge (OR 4.759, CI 95% 2.106–10.753; *p* = 0.000). These results show a good level of awareness about COVID-19 vaccination in this population, which may indicate the effectiveness of communication strategies accompanying the COVID-19 immunization campaign in Italy.

## 1. Introduction

Coronavirus disease 2019 (COVID-19) is a severe acute respiratory disease (SARS) caused by the novel coronavirus (SARS-COV-2) first detected in Wuhan, China, at the end of 2019 [1]. Since its onset, SARS-COV-2 has spread rapidly throughout the world [2], having a significant impact on healthcare services and workers [3]. Currently, the COVID-19 pandemic has registered approximately 154 million cases and more than 3 million deaths [2]. Due to this social and healthcare burden, countries have adopted several strategies to control the spread of the virus, including social distancing, suspension or modification of working activities, restricted movement, and obligatory use of facial masks [4]. Although necessary, these measures have had indirect negative consequences on national economies and personal health, leading to a rise in mental disorders and unhealthy behaviors [5,6,7,8,9,10].

In 2020, efforts were made across the world to rapidly develop vaccines for COVID-19 [11]. In this process, scientific and medical communities worked together with governmental institutions, with the result that several effective vaccines were approved in record time [11,12]. At the end of 2020, the Italian Ministry of Health launched the strategic immunization plan, aimed at achieving herd immunity in the Italian population [13,14,15]. Health and teaching personnel, police forces, and elderly and vulnerable people were prioritized for vaccinations [13,14,15]. At the beginning of 2021, three vaccines began to be administered in Italy: the mRNA vaccines Moderna (by Moderna Biotech) and Comirnaty (by Pfizer/BioNTech), and the viral vector vaccine Vaxzevria (by AstraZeneca) [13,14,15]. Since that time, the effort to achieve herd immunity throughout the Italian territory has involved a notable economic and organizational effort by Italian institutions and authorities. However, as vaccination is voluntary, popular acceptance represents a key factor.

Vaccine hesitancy, which consists of a delay in acceptance or a refusal of vaccination despite the availability of vaccination services [16], may compromise the success of any immunization campaign. This is particularly critical in Italy, where, over the last decade, vaccine hesitancy movements have led to a growing mistrust of vaccines and a subsequent decline in vaccination coverage rates, forcing the Italian Ministry of Health to increase the number of mandatory infant vaccines [17]. Furthermore, both the accelerated development of the COVID-19 vaccines [18] and the novel formulation of some of these vaccines [19] may have contributed to increasing vaccine hesitancy. On March 15 2021, the Italian Drug Agency (Agenzia Italiana del Farmaco (AIFA)) decreed the precautionary suspension of the Vaxzevria vaccine following some cases of thrombosis registered worldwide and in Italy [20]. While this suspension was revoked three days later [21], the event may have nonetheless increased vaccine hesitancy. Recent studies have underlined that different vaccine sentiments may create pockets of unvaccinated subjects, even in large communities of vaccinated people [22]. This represents a critical issue for public health management, as it can increase the risk for vulnerable individuals living within these unvaccinated pockets.

Since vaccine hesitancy strictly relates to level of knowledge and sources of information, it is important to measure vaccine acceptance among various segments of the population in order to develop effective communication strategies against misinformation [19,23]. Furthermore, age seems to play an important role in determining vaccine acceptance, with contrasting effects in different countries [18]. Therefore, the analysis of vaccine acceptance and its predictors in different age classes may be helpful for identifying hesitant groups and addressing their specific fears and concerns. With respect to Italian young adults, a study performed by Barello et al. in 2020, prior to the national immunization campaign, reported a preliminary intention to be vaccinated against COVID-19 in 86.1% of the sample [24].

In order to analyze the evolution of vaccine hesitancy in this target population, the present study aimed at appraising knowledge about and acceptance of COVID-19 vaccination in a sample of Italian undergraduates during the first phase of the national immunization campaign. 

## 2. Materials and Methods

### 2.1. Setting and Participants

This cross-sectional study was conducted in the period from February to April 2021. Participants were Italian undergraduate students attending three Italian universities in Rome, Naples, and Bari, respectively. During the study period, the selected universities were providing lessons via the Internet due to the control measures in place during the second wave of the COVID-19 pandemic. Students were invited during remote lessons to voluntarily participate in the survey by responding to an online questionnaire. The survey was carried out simultaneously in the three universities.

In total, the three universities had 158,462 students in the target population of undergraduates; thus, a sample of at least 384 individuals was required to explore the selected variables, assuming a response rate of 50%, a 95% confidence level, and a 5% margin of error, as discussed in previous studies [6,7].

The study was performed in accordance with the World Medical Association Declaration of Helsinki. Participants were guaranteed the anonymity of the collected information. The Scientific and Ethical Institutional Board of the Italian Inter University Research Centre “Population, environment and health” (CIRPAS) approved the protocol (approval number 2101_2021).

### 2.2. Questionnaire

The survey was comprised of an original questionnaire written in the Italian language and administered using SurveyMonkey. The questionnaire included three sections.

The first section aimed at collecting sociodemographic information (i.e., gender, age, university, degree course, parents’ educational level, whether or not they were a healthcare worker) and personal experience of COVID-19 (personal infection/infection of a relative; infection that was asymptomatic/symptomatic/severe). These items were designed on the basis of a previous study with the same student population [6] and the advice of a panel of experts comprised of one demographer, one epidemiologist, and one psychologist.

The second section of the questionnaire investigated respondents’ general acceptance of vaccination and specific acceptance of the COVID-19 vaccine. This section was modeled on the questionnaire used by Sallam et al. [25], which was adapted to the Italian context. The modified items were drafted by a panel of experts comprised of two epidemiologists, one expert in public health, and one psychologist. Respondents were asked to declare if they were favorable to vaccinations in general (yes/no/I don’t know), if they had been immunized against the influenza virus in 2019/20 and/or 2020/21 (yes/no), if they had received at least a single dose of the COVID-19 vaccine (yes/no), and if they were willing to be vaccinated against COVID-19 (yes/no/I don’t know). A further question was posed to those who declared their unwillingness to be vaccinated in order to obtain their main motivations for this position (I don’t trust vaccines/these vaccines are not effective/I’m allergic/I’ve had the disease/I’m not at risk). 

The third section aimed at assessing respondents’ knowledge, opinions, and sources of information about the COVID-19 vaccines. This section was based on available data regarding COVID-19 vaccination issues and vaccination hesitancy [11,12,25], as well as statements issued by national institutions [13]. Items in this section were drafted by a panel of experts comprised of one epidemiologist, one expert in vaccinology, and one psychologist. The items investigated in this section are reported in the Supplementary Materials (File S1).

Prior to its administration in the present study, the questionnaire was tested in a pilot study (data neither published nor included in this paper). Overall, 144 students were enrolled for this preliminary study. In order to evaluate the comprehensibility of the questions, students were asked to assign a score to each question on a seven-point scale ranging from 1 (*not meaningful*) to 7 (*very meaningful*). Moreover, as discussed in previous studies [6,7], in order to guarantee variability in the answers for the pilot study, 12 further questions (FQs) including errors (grammatical and/or semantic) were added to the original questionnaire questions (OQs). The OQs resulted in a mean score for each question of ≥6 (almost the maximum); the FQs produced a mean score of <2. These data confirmed that the content of the questionnaire was clear. The reliability index was assessed for both the pilot and the original questionnaire using Cronbach’s alpha (internal consistency coefficient) [26,27]. The alpha values were 0.83 and 0.79, respectively, showing a satisfactory level of reliability [28].

### 2.3. Statistical Analyses

A descriptive analysis was performed on participants’ demographic characteristics and examined experience, acceptance, knowledge, and opinions about COVID-19 vaccination. Age was expressed as a mean value ± standard deviation (*SD*). Other characteristics and answers were reported as numbers and percentages of respondents. Influenza vaccination rates reported for the seasons 2019/20 and 2020/21 and answers regarding willingness to be vaccinated and consequences of COVID-19 vaccination on health, registered before and after the suspension of Vaxzevria on March 15 2021, were compared using a chi-squared test. Differences in vaccine acceptance among the three university groups were analyzed through the chi-squared test. Level of knowledge was expressed as the total number of correct answers (range 0–11). The median and the interquartile range were calculated for knowledge. The median number of correct answers from the three participant universities was compared through the Kruskal–Wallis test. A Spearman correlation analysis was conducted to highlight possible relationships between the level of knowledge of participants and variables related to their sociodemographic characteristics and vaccine acceptance. A logistic regression analysis was performed to investigate the possible role of sample characteristics and knowledge about vaccination in determining COVID-19 vaccine acceptance. In particular, gender (expressed as male = 0; female = 1), age (lower or equal to median value = 0; higher than median value = 1), area of study (other = 0; life science = 1), parents’ educational level (elementary/middle school = 0; high school = 1; degree/post-degree = 2), whether or not the participant was a healthcare worker (no = 0; yes = 1), personal experience of COVID-19 (no = 0; yes = 1), and level of knowledge (number of correct answers lower or equal to median value = 0; higher than median value = 1) were considered as independent variables. Vaccine acceptance was considered as the dependent variable, with a value of 0 attributed to respondents who had not yet been vaccinated against COVID-19 and were not willing to be immunized and a value of 1 attributed to those who had been vaccinated or were favorable to vaccination. A preliminary univariate analysis was performed to detect variables significantly associated with vaccine acceptance; subsequently, these variables were included in the logistic regression analysis. Results were expressed as odds ratios (ORs) with a corresponding 95% confidence interval (95% CI). A *p*-value of 0.05 was assumed as a significance level.

The software IBM SPSS version 27 for Windows (IBM Corp., Armonk, NY, USA) was used for all analyses.

## 3. Results

A total of 3226 students completed the questionnaire in full. Table 1 shows the sociodemographic characteristics of the sample.

The majority of the sample was composed of females and students attending a life science course, and mainly a high school educational level was reported for both parents. A small proportion of participants (8.2%) were healthcare workers. The great majority of the sample had not been infected with COVID-19, while approximately 30% reported an infected relative. For these relatives, a symptomatic form of the disease was mainly reported.

Table 2 presents participants’ expressed acceptance of vaccination, in general, and COVID-19 vaccination, in particular.

Nearly all participants were favorable to vaccination in general. Although the majority of the sample had not been vaccinated against influenza (during the prior two seasons) or COVID-19, more than 90% declared their willingness to be vaccinated against COVID-19. Interestingly, the proportion of students who had been immunized against influenza more than doubled in 2020/21 relative to the previous season, and the difference in vaccination between the two seasons was statistically significant (*p* < 0.001).

Table 3 presents participants’ knowledge and opinions about COVID-19 vaccination.

With respect to the characteristics of the available COVID-19 vaccines, approximately 64% of the sample knew that they contained the genetic information for viral antigen production, and approximately 96% were aware that they should be administered in two doses. Roughly 80% answered correctly that the influenza vaccine does not prevent COVID-19; that the COVID-19 vaccines may prevent and do not cause the disease, may reduce symptoms, and do not act through human DNA modification; that COVID-19 vaccination does not negate the need to engage in other prevention measures; that the COVID-19 vaccines should not only be offered to elderly people and healthcare personnel; and that herd immunity will not be reached through only the immunization of these latter populations. Finally, 62.5% thought that all of the Italian population need to be vaccinated against COVID-19, and 59.5% felt that COVID-19 vaccination should be compulsory. The median number of correct answers for the whole sample was 9 (interquartile range 8–10).

Approximately half of the sample perceived that the COVID-19 vaccines might cause some health effects, while more than 95% did not consider vaccination a risk to privacy. A significant increase (from 24.3% to 69.7%, *p* < 0.001) in positive answers to the question regarding the health consequences of the COVID-19 vaccines was registered after the suspension of Vaxzevria. Nonetheless, this event did not impact on respondents’ willingness to be vaccinated, which showed a significant increase (from 88.8% to 95.2%, *p* < 0.001). 

Healthcare personnel/scientists and the mass media were reported as the main sources of information. 

**Figure 1:**
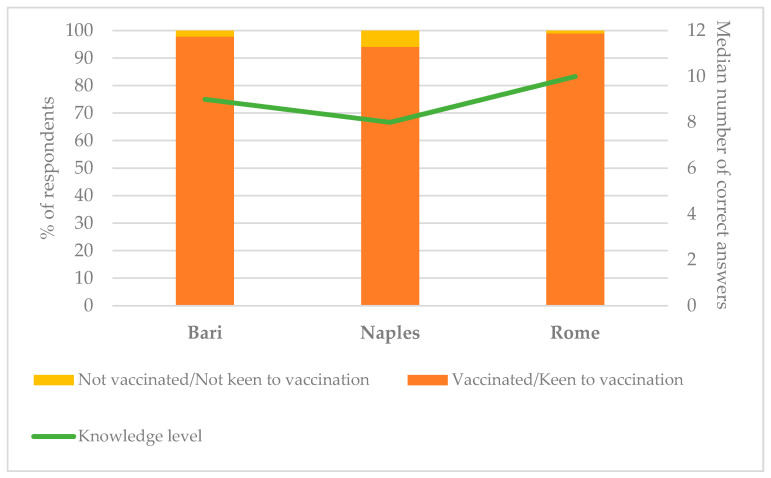
Acceptance (percentage of students vaccinated/keen to be vaccinated) and knowledge (median number of correct answers) regarding COVID-19 vaccination in the three participant universities.

As for the comparison among the three universities, Figure 1 shows the proportions of students vaccinated/keen to be vaccinated and the median level of knowledge in the three undergraduate groups.

Both the acceptance of COVID-19 vaccination and the level of knowledge were lower in participants attending the University of Naples than in the other groups. Both these differences were significant (*p* < 0.001).

Table 4 shows the results of the correlation analysis performed on the variables.

A significant correlation with knowledge level was found for all of the considered variables; in particular, age, parents’ educational level, attending a life science course, being a healthcare worker, and being vaccinated or keen to be vaccinated against COVID-19 were positively correlated with knowledge. On the contrary, female gender and previous experience of COVID-19 were negatively correlated with knowledge.

Table 5 reports the results of the logistic regression analyses performed including only those independent variables that were significantly associated with vaccine acceptance in the univariate analysis: age class (lower than or equal to/higher than the median value of 22 years), gender (male/female), previous influenza vaccination (no/yes), and level of knowledge about COVID-19 vaccination (number of correct answers lower than or equal to/higher than the median value of 9).

Being vaccinated against influenza in 2019/20 (*p* = 0.025) and a higher level of knowledge (*p* = 0.000) were found to be associated with being vaccinated/keen to be vaccinated against COVID-19.

## 4. Discussion

The present study aimed at assessing knowledge and acceptance of COVID-19 vaccination among Italian undergraduates. The results show a high level of acceptance towards COVID-19 vaccination and a good level of knowledge regarding the vaccine characteristics and immunization campaign in this population group. Moreover, knowledge and vaccine acceptance were found to be correlated.

With respect to knowledge, the only critical issue was registered with regard to the formulation of the available vaccines. However, it should be noted that approximately 45% of the sample was comprised of students attending courses outside of the life sciences, and this may have determined their incomplete comprehension of how the new vaccines work. As for the perception of negative health consequences from the vaccines, this increased significantly after the precautionary suspension of Vaxzevria. At the same time, surprisingly, acceptance of COVID-19 vaccination also increased significantly. A potential explanation for this is that the sample was more worried about COVID-19 than a possible adverse effect of vaccination. Such an attitude might have been determined by the sources of information consulted: fewer than 10% of respondents acquired information from uncontrolled sources (social media, web channel, etc.).

Greater knowledge about COVID-19 vaccination was related to older age, male gender, higher educational level of parents, being a healthcare worker, attending a life science course, and acceptance of COVID-19 vaccination. On the contrary, female gender and direct experience with the disease were inversely related to vaccine knowledge. The finding related to gender contrasts with previous findings of better knowledge among female students [6]; this is likely due to the higher male/female ratio in the present study, denoting a better sample distribution by gender.

The proportion of respondents willing to receive COVID-19 vaccination (91.9%) was higher than that registered in another Italian region (Emilia Romagna) by Reno et al. in January 2021 (68.9%) [29] and by Barello et al., who reported a low intention to vaccinate (vaccine hesitancy) among 13.9% of their interviewed students [24]. However, the present finding is in line with the 91% vaccine acceptance reported by Biasio et al. in their study performed during the same months on a sample of Italian adults [30]. Notably, in the present study, the high acceptance of COVID-19 vaccination was not influenced by knowledge regarding vaccine effectiveness: only 80% of the sample thought that the vaccine was effective in preventing the disease. Regardless, the sample considered the vaccine helpful in reducing the chance of infection.

In Barello et al.’s study, no differences in vaccine acceptance were found between students attending healthcare and non-healthcare degree courses [24]. On the contrary, the present study found greater knowledge about COVID-19 and greater willingness to receive the vaccine among life science undergraduates, as also demonstrated in a previous study [6]. This finding is notable considering the potential risks linked to healthcare students’ vaccine hesitancy: vaccination of healthcare workers and students is a key measure in the prevention of healthcare-associated COVID-19 infections due to the close contact of these populations with high-risk patients. For this reason, public health information campaigns should aim at raising awareness of the crucial role of individual control measures, such as vaccination, in safeguarding individual and community health [24].

Our analysis made it possible to highlight differences in the outcomes among the universities enrolled, with students from Naples showing lower acceptance and knowledge levels. This aspect should be further investigated in depth with a specific protocol targeted at analyzing possible differences related to locations.

The regression analysis showed that both knowledge and vaccination against influenza in 2019/20 were related to vaccine acceptance. This is consistent with findings from another study showing that prior vaccination against seasonal influenza predicted intention to be immunized against COVID-19 [31].

Notably, in the multivariable analysis, age and gender were not significantly associated with vaccine acceptance, though females were significantly less favorable to vaccination than males (97.7% vs. 98.8%, *p* = 0.016). This finding contrasts with the results of other studies showing a lower intention to be vaccinated against COVID-19 in women and younger adults [29,31,32,33]. The age range of our sample was potentially too narrow to detect age differences. Furthermore, personal infection with COVID-19 did not emerge as a significant predictor of vaccine willingness and acceptance, as reported by Reno et al. [29]. Most likely, respondents who had already experienced the disease considered themselves immune against a new infection. In contrast, experience of a relative being infected with COVID-19 was inversely correlated with knowledge, suggesting that a low level of knowledge may have led respondents to ignore the control measures, thereby increasing their risk of contracting the disease.

With regard to influenza vaccination, other authors have reported a similar percentage of vaccinated students (38.6%) [30]. Moreover, in the present study, the reported vaccination rate in 2020/21 was significantly higher than that of the previous season. This suggests that the COVID-19 pandemic may have increased attention to other SARS agents and raised respondents’ willingness to receive the influenza vaccine [34].

The present study has some limitations. First, knowledge could not be investigated in depth due to the need to avoid an excessive length of the questionnaire. This could have hidden important information regarding uncollected variables. Furthermore, the study population was enrolled by convenience sampling from three Italian universities; these students represent a specific population group and are not representative of the whole population of young adults in Italy. Moreover, it should also be taken into account that our sample only included undergraduates attending universities located in central and southern Italy, with the exclusion of northern ones. Considering the important differences in the socio-economic characteristics of northern, central, and southern areas in the Italian territory, and also the varying impact of the COVID-19 epidemic among these areas, a comparison with other undergraduate groups from northern regions would have been useful to detect possible geographical differences.

However, the study offers a picture of vaccine acceptance and knowledge in a large sample of Italian undergraduates during the first phase of the COVID-19 immunization campaign. It confirms that knowledge and acceptance are strictly related, underlining the role of correct information in fighting vaccine hesitancy [19,23]. This issue should be continually monitored and characterized over time.

## 5. Conclusions

Notwithstanding the enrollment of universities located only in central and southern Italy, our study found high knowledge and acceptance of COVID-19 vaccination among Italian undergraduates. This supports the effectiveness of the information strategy that has accompanied the COVID-19 immunization campaign in Italy. Nonetheless, the situation requires constant monitoring. Policy makers, government officials, and the media should pay attention to the spread of data not supported by scientific evidence that may affect vaccine acceptance.

## Figures and Tables

**Table 1:** Sample characteristics.

Variable	Participants *n =* 3226
Age, years	
Mean ± *SD*	23.3 ± 3.9
Range	18–45
Median value	22
Interquartile range	21–25
Gender, *n (%)*	
Male	1421 (44)
Female	1805 (56)
Father‘s educational level, *n (%)*	
Elementary/middle school	846 (26.2)
High school	1596 (49.5)
Degree/post-degree	784 (24.3)
Mother‘s educational level, *n (%)*	
Elementary/middle school	755 (23.4)
High school	1603 (49.7)
Degree/post-degree	868 (26.9)
Area of study, *n (%)*	
Life science	1787 (55.4)
Other	1439 (44.6)
Healthcare worker, *n (%)*	
Yes	264 (8.2)
No	2962 (91.8)
COVID-19 infection, *n (%)*	
Yes	299 (9.3)
Asymptomatic	72 (24.1)
Symptomatic	219 (73.2)
Severe	8 (2.7)
No	2927 (90.7)
COVID-19 infection in relative, *n (%)*	
Yes	947 (29.4)
Asymptomatic	143 (15.1)
Symptomatic	602 (63.6)
Severe	132 (13.9)
Dead	70 (7.4)
No	2279 (70.6)

**Table 2:** Participants’ acceptance of general vaccination and COVID-19 vaccination.

Question	Respondents *n =* 3226
Favorable to vaccination, *n (%)*	
Yes	3012 (93.4)
No	115 (3.6)
I don’t know	99 (3.1)
Vaccinated against influenza (2019/20 season), *n (%)*	
Yes	515 (16)
No	2711 (84)
Vaccinated against influenza (2020/21 season), *n (%)*	
Yes	1244 (38.6)
No	1982 (61.4)
Vaccinated against COVID-19, *n (%)*	
Yes	375 (11.6)
No	2851 (88.4)
Willing to be vaccinated against COVID-19, *n (%)*	
Yes	2621 (91.9)
No	55 (1.9)
I don’t know	175 (6.1)

**Table 3:** Participants’ knowledge and opinions about COVID-19 vaccination.

Question	Respondents *n =* 3226
The available COVID-19 vaccines contain:	
The coronavirus	122 (3.8)
A virus similar to coronavirus	53 (1.6)
The antigens of the virus (the protein “spike”)	994 (30.8)
The genetic information to build the antigen “spike”	2057 (63.8)
The available COVID-19 vaccines are administered:	
In a single dose	60 (1.9)
In two doses	3095 (95.9)
In two doses only for those subjects who are not immunized with the first administration	71 (2.2)
Do you think that influenza vaccination may protect against COVID-19? *n (%)*	
Yes	707 (21.9)
No	2519 (78.1)
Do you think that the COVID-19 vaccines are effective in preventing COVID-19 infection? *n (%)*	
Yes	2598 (80.5)
No	628 (19.5)
Do you think that the COVID-19 vaccines may reduce symptoms of COVID-19? *n (%)*	
Yes	2527 (78.3)
No	699 (21.7)
Do you think that the COVID-19 vaccines cause the disease in order to trigger immunity? *n (%)*	
Yes	626 (19.4)
No	2600 (80.6)
Do you think that the COVID-19 vaccines modify the DNA of vaccinated subjects? *n (%)*	
Yes	316 (9.8)
No	2910 (90.2)
Do you think that people vaccinated against COVID-19 can avoid other prevention measures, such as facial masks? *n (%)*	
Yes	119 (3.7)
No	3107 (96.3)
Do you think that all of the Italian population need to be vaccinated against COVID-19? *n (%)*	
Yes	2015 (62.5)
No	1211 (37.5)
Do you think that only health personnel and elderly people need to be vaccinated against COVID-19? *n (%)*	
Yes	112 (3.5)
No	3114 (96.5)
Do you think that “herd immunity” will be reached in Italy when all health personnel and elderly people are vaccinated against COVID-19? *n (%)*	
Yes	231 (7.2)
No	2995 (92.8)
In your opinion, might the COVID-19 vaccines cause health problems? *n (%)*	
Yes	1514 (46.9)
No	1712 (53.1)
In your opinion, might the COVID-19 vaccines negatively impact on individual privacy? *n (%)*	
Yes	159 (4.9)
No	3067 (95.1)
In your opinion, should COVID-19 vaccination become mandatory? *n (%)*	
Yes	1921 (59.5)
No	1305 (40.5)
What are your main sources of information about COVID-19 vaccination? *n (%)*	
Healthcare personnel, scientists	1434 (44.5)
Mass media (i.e., television, general interest magazines)	1503 (46.6)
Social media (i.e., Facebook, Twitter, Instagram, WhatsApp)	268 (8.3)
YouTube or similar web channel	21 (0.7)

**Table 4:** Results of the correlation analysis between knowledge about COVID-19 vaccination and other variables.

Variable	Spearman’s Correlation Coefficient *p*-Value
Age	0.271 *0.000*
Gender	−0.160 *0.000*
Father‘s educational level	0.178 *0.000*
Mother‘s educational level	0.203 *0.000*
Area of study	0.228 *0.000*
Healthcare worker	0.036 *0.039*
COVID-19 infection	−0.072 *0.000*
COVID-19 infection in relative	−0.070 *0.000*
Vaccinated/keen to be vaccinated against COVID-19	0.079 *0.000*

**Table 5:** Results of the logistic regression model built considering the acceptance of COVID-19 vaccination as the outcome.

Independent Variable	Vaccine Acceptance OR (CI 95%)
Age	
≤22 years	Reference
≥23 years	0.774 (0.447−1.341)
Gender	
Male	Reference
Female	0.597 (0.329–1.083)
Vaccinated against influenza (season 2019/20)	
No	Reference
Yes	3.806 (1.181–12.267) *
Level of knowledge about COVID-19 vaccines	
≤9 correct answers	Reference
≥10 correct answers	4.759 (2.106–10.753) **

OR (CI 95%): odds ratio (95% confidence interval); * *p* < 0.05; ** *p* < 0.01.

## Data Availability

All data presented are available upon request from the corresponding author (F.G.).

## Supplementary Materials

The following are available online at https://www.mdpi.com/article/10.3390/vaccines9060638/s1, Supplementary File 1 (S1): Questionnaire section regarding knowledge and opinions on COVID-19 vaccines.
