# Supplementary material for: Knowledge and Acceptance of COVID-19 Vaccination among Undergraduate Students from Central and Southern Italy
Source: Vaccines (Basel). 2021 Jun 10;9(6):638. doi: 10.3390/vaccines9060638 (PMC8230551; doi:10.3390/vaccines9060638)
Supplement: Supplementary file 1 [file vaccines-09-00638-s001.zip › Supplementary File 1.pdf]

Article

Knowledge and Acceptance of COVID-19 Vaccination among Undergraduate Students from Central and Southern Italy

Questionnaire section regarding knowledge and opinions on COVID-19 vaccines

- The available COVID-19 vaccines contain:

the coronavirus
a virus similar to coronavirus
the antigens of the virus (the protein "spike")
the genetic information to build the antigen "spike"

- The available COVID-19 vaccines are administered:

in a single dose
in two doses
in two doses only for those subjects who are not immunized with the first

- Do you think that influenza vaccination may protect against COVID-19?

yes/no

- Do you think that the COVID-19 vaccines are effective in preventing COVID-19 infection?

yes/no

- Do you think that the COVID-19 vaccines may reduce symptoms of COVID-19?

yes/no

- Do you think that the COVID-19 vaccines cause the disease in order to trigger immunity?

yes/no

- Do you think that the COVID-19 vaccines modify the DNA of vaccinated subjects?

yes/no

- Do you think that people vaccinated against COVID-19 can avoid other prevention measures, such as facial masks?

yes/no

- Do you think that all of the Italian population need to be vaccinated against COVID-19?

yes/no

- Do you think that only health personnel and elderly people need to be vaccinated against COVID-19?

yes/no

- Do you think that "herd immunity" will be reached in Italy when all health personnel and elderly people are vaccinated against COVID-19?

yes/no

- In your opinion, might the COVID-19 vaccines cause health problems?

yes/no

- In your opinion, might the COVID-19 vaccines negatively impact on individual privacy?

yes/no

- In your opinion, should COVID-19 vaccination be mandatory?

yes/no

- What are your main sources of information about COVID-19 vaccination?

health care personnel, scientists
mass media (i.e., television, general interest magazines)
social media (i.e., Facebook, Twitter, Instagram, WhatsApp)
YouTube or a similar web channel
